# RLIP76, a non-ABC transporter, and drug resistance in epilepsy

**DOI:** 10.1186/1471-2202-6-61

**Published:** 2005-09-27

**Authors:** Sanjay Awasthi, Kerri L Hallene, Vince Fazio, Sharad S Singhal, Luca Cucullo, Yogesh C Awasthi, Gabriele Dini, Damir Janigro

**Affiliations:** 1 Department of Chemistry and Biochemistry, University of Texas at Arlington, Arlington, TX USA; 2 Cerebrovascular Research, Department of Neurological Surgery, Cleveland Clinic Foundation, Cleveland, OH USA; 3 Molecular Medicine, Cleveland Clinic Lerner College of Medicine, Cleveland, OH USA; 4 Department of Human Biological Chemistry and Genetics, University of Texas Medical Branch at Galveston, Galveston, TX USA

## Abstract

**Background:**

Permeability of the blood-brain barrier is one of the factors determining the bioavailability of therapeutic drugs and resistance to chemically different antiepileptic drugs is a consequence of decreased intracerebral accumulation. The ABC transporters, particularly P-glycoprotein, are known to play a role in antiepileptic drug extrusion, but are not by themselves sufficient to fully explain the phenomenon of drug-resistant epilepsy. Proteomic analyses of membrane protein differentially expressed in epileptic foci brain tissue revealed the frequently increased expression of RLIP76/RALBP1, a recently described non-ABC multi-specific transporter. Because of a significant overlap in substrates between P-glycoprotein and RLIP76, present studies were carried out to determine the potential role of RLIP76 in AED transport in the brain.

**Results:**

RLIP76 was expressed in brain tissue, preferentially in the lumenal surface of endothelial cell membranes. The expression was most prominent in blood brain barrier tissue from excised epileptic foci. Saturable, energy-dependent, anti-gradient transport of both phenytoin and carbamazepine were demonstrated using recombinant RLIP76 reconstituted into artificial membrane liposomes. Immunotitration studies of transport activity in crude membrane vesicles prepared from whole-brain tissue endothelium showed that RLIP76 represented the dominant transport mechanism for both drugs. RLIP76^-/- ^knockout mice exhibited dramatic toxicity upon phenytoin administration due to decreased drug extrusion mechanisms at the blood-brain barrier.

**Conclusion:**

We conclude that RLIP76 is the predominant transporter of AED in the blood brain barrier, and that it may be a transporter involved in mechanisms of drug-resistant epilepsy.

## Background

Each year approximately 160,000 persons are diagnosed with epilepsy, and about 10,000 of these individual develop drug-resistant epilepsy [1]. The causes of drug-resistant epilepsy are numerous, many due to ontogenic abnormalities in brain maturation, severe brain injuries with resultant irreversible changes to cerebral neuroglial organization and inhibitory neuron function, kindling phenomenon, seizure-induced disturbances of oxygen supply, as well as acquired (or hereditary) changes in transporter proteins of the blood-brain barrier which function in the efflux of anti-epileptic drugs (AEDs) from the brain. The latter mechanisms have been the focus of intense efforts to develop new rationally designed AEDs that could bypass these transport mechanisms. Unfortunately, the identity of the multiple transport mechanisms of the blood brain barrier, and the individual role of each in mediating drug-resistant epilepsy is as yet incompletely defined.

The ABC-family transporters have been the subject of considerable interest in the mechanisms of drug-resistant epilepsy [2,3]. The prototypical ABC transporter, P-glycoprotein (Pgp, MDR1, or mdr1 gene product), as well as MRP2, and BCRP are expressed in the blood-brain barrier [2]. Endothelial expression of Pgp has been demonstrated, and the role of Pgp in regulating brain drug-concentrations has been established in knockout mouse studies [4]. These studies evaluated the transport of a number of centrally acting drugs including antipsychotics, ant emetics, and natural product antineoplastic agents. The only AED examined, phenytoin (PHE), appeared to be a weak substrate of Pgp in intact cell transport experiments. Carbamazepine (CBZ) has been found not to inhibit Pgp mediated transport, thus would be a poor substrate [5]. Recently, studies examining the ability of AED to competitively inhibit the transport activity of Pgp have found little effect of AEDs on Pgp activity, and then only at concentrations well above clinically achieved therapeutic levels [6]. Although Pgp appears to mediate some minor transport activity towards most CNS active agent, the greatest CSF/plasma ratios (6.6–17 fold) were seen with antipsychotics and antiemetics rather than AEDs, evidence has been presented which suggests that there have been no reports directly demonstrating transport of any AED by Pgp in an isolated system [7].

In epileptic patients, expression of MDR1 has a complex pattern that does not directly support a significant pharmacokinetic role for MDR1 in human epilepsy since MDR1 expression was found in both blood-brain barrier and parenchymal cells in epileptic brain [8-10]. This is based on the possibility that expression of MDR1 in glia may actually favor drug interactions with neuronal by reducing the accumulation of drug in the glial syncitium. Conversely, parenchymal expression may shift concentrations in the extracellular and intracellular space or may mediate compartmentalization, and thus may reduce concentrations of antiepileptic drugs at their target sites. The fact that MDR1 may not be a crucial determinant for multiple drug resistance to antiepileptic drugs has been recently challenged in a number of reports [11-13]. Other ABC-transporters, most notably MRP2 and BCRP have also been localized to the blood-brain barrier, but similarly, direct evidence for the ability of these proteins to transport AEDs in isolated systems has been lacking. Because of the lack of information regarding the kinetic parameters of transport, the relative contributions of different ABC transporters in mediating drug-resistant epilepsy remains unknown and a major impediment to development of novel targeted anti-epileptic agents.

We have recently described a novel non-ABC mutispecific transporter, RLIP76, a multifunctional modular protein found ubiquitously from Drosophila to humans [14-17]. It is encoded in humans on chromosome 18p11.3 by a gene with 11 exons and 9 introns. RLIP76 is a 76 kDa protein product of this gene, but splice-variants including a 67 kDa peptide and longer 80 or 102 kDa peptide, cytocentrin have been identified [18]. RLIP76 was cloned as a Ral-binding protein and predicted to be an effector involved in regulation of membrane plasticity, movement, and endocytosis [17,19-21]. RLIP76 was identified as a highly active efflux mechanisms for removing glutathione-electrophile conjugate (GS-E, i.e. LTC4) from cells [22]. In addition to GS-E, the exceptionally broad substrate specificity of RLIP76 extends to Pgp substrates including anthracycline and vinca alkaloids, towards which it mediates resistance [23-27]. Studies on proteomic screening of epileptic foci led to the identification of RLIP76 as being frequently up-regulated. Present studies were carried out to examine the role of RLIP76 in AED transport in the blood-brain barrier.

## Results and discussion

**Figure 1:**
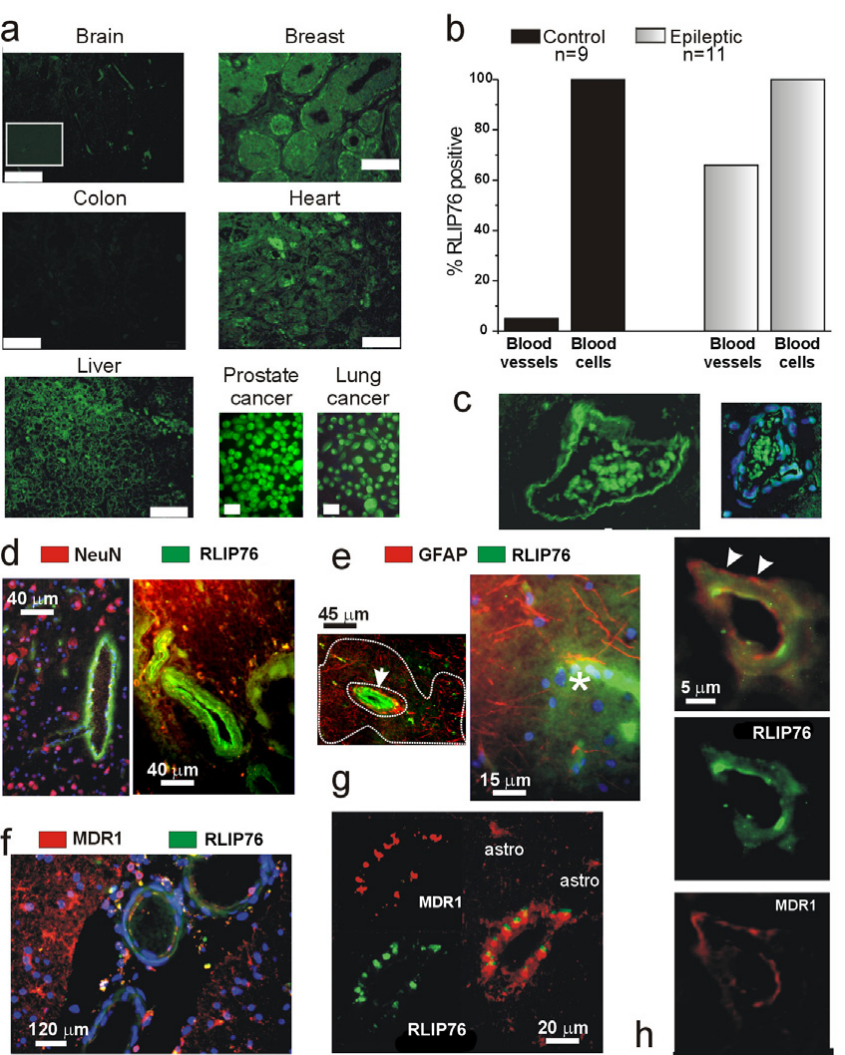
**Human expression of RLIP76. A) **Immunocytochemical detection was performed on tissue arrays with histological sections from normal human tissue. RLIP76 expression was virtually absent from normal brain autopsies; both gray and white matter were analyzed; cumulative data for brain and blood tissue are presented in **b**). RLIP76 immunoreactivity was observed in breast lobules of primary duct elements, in cardiac myocytes and liver sinusoids. No expression was found in colon tissue. Intense immunoreactivity was present in cell lines of tumor origin. Bars indicate 60 μm with the exception of the cell culture data, where the bars reflect 10 μm. The inset in **a) **shows a negative control of a brain section incubated with secondary but not primary antibodies. **B) Cerebrovascular expression of RLIP76 in epileptic brain. **Blood vessels from multiple drug resistant epileptic brain were characterized by high levels of RLIP76 expression. Both vascular and intravascular cells were RLIP76 immunopositive. RLIP76-positive intravascular cells were anucleated and did not react with the nuclear stain DAPI (in *blue*, **C**). Note that erythrocytes were found to be RLIP76 immunopositive in both normal and epileptic tissue. **RLIP76 is expressed exclusively in epileptic endothelial cells and does not localize to glia or neurons. D) **NeuN expression is segregated from RLIP76 immunoreactivity, which is limited to cortical vessels. **E) **Widespread GFAP immunoreactivity in epileptic brain does not co-localize with RLIP76 (in *green*). Both large (*arrow*) and capillary-size (*asterisk*) vessels express RLIP76. Note the large region of GFAP positive reactive gliosis (limited by a *dotted line*) characterized by the absence of RLIP76 expression. **F) MDR1 and RLIP76 co-localize in "epileptic" blood vessels but MDR1 expression extends to parenchymal cells. **Three large pial vessels are shown to demonstrate the predominant vascular expression of RLIP76. Note that MDR1 expression was more predominant in parenchymal glia. **G-H) High power demonstration of endothelial co-expression of RLIP76/MDR1. **Note that MDR1 expression co-localized with RLIP76 expressed at the lumenal surface, while MDR1 expression was also observed in RLIP76-negative ablumenal structures reflecting glial endfeet (*arrowheads *in H). We studied a total of 41 patients, including samples from 6 autopsies. The average age of the patients was 32 ± 18 years, range 3 months – 59 years old. No data are available on the autopsy material, besides the fact that these were adults of either sex. The non-epileptic patients were either undergoing surgery for aneurysm clipping or to remove arteriovenous or other vascular malformations. None of these patients had seizures prior to surgery or received antiepileptic drug treatment. The surgical epileptic patients studied were resistant to the following drugs: CBZ = 61%; PHE = 77%; Pentobarbital = 55%; Tompiramate = 50%; valproic acid = 44%; other AEDs, less than 5%. Most patients were resistant to > two drugs; 1 patients (a 3 months old infant) did not undergo any drug treatment, while another patient (8 month old) attempted ketogenic diet treatment).

RLIP76 was expressed in all normal human tissues examined, more prominently in breast, heart, liver and erythrocytes, and less so in colon and brain parenchyma (Fig. 1a, b &1c). Significant expression was seen in malignant human cell lines including the PC-3 human prostate cancer and H1299 human non-small cell lung cancer cell lines. Whereas RLIP76 was barely detectable in normal brain parenchyma or vessels, blood vessels from epileptic patients has a markedly increased expression of RLIP76 (Fig. 1b &1c).

Epileptic brain sections obtained from multiple drug resistant patients also revealed substantial differences compared to non-epileptic brain. By immunocytochemical analysis, we discovered widespread RLIP76 expression in the cerebral vasculature from epileptic brain. We used double label immunofluorescence to reveal that RLIP76 co-localized with the multidrug resistance transporter P-glycoprotein (MDR1) but not with NeuN or GFAP (Fig. 1d, e, f, and 1h). However, while MDR1 was expressed in both parenchymal and endothelial cells [8,9], RLIP76 immunoreactivity was limited to the vasculature. No overlapping expression of RLIP76 was observed in GFAP positive astroglia (Fig. 1e) or NeuN positive neurons (Fig. 1d). By confocal analysis, RLIP76 expression was found in capillary endothelial cells, penetrating pial vessels, and larger (>100 μm) vessels (Fig. 1d, e, and 1f). In capillary endothelial cells, MDR1 expression was both lumenal (endothelial) and abluminal (glial endfeet), whereas RLIP76 expression appeared to be predominantly lumenal and did not co-localize with GFAP immunoreactivity (Fig. 1g and 1h). Additional studies are needed to confirm this finding.

**Figure 2:**
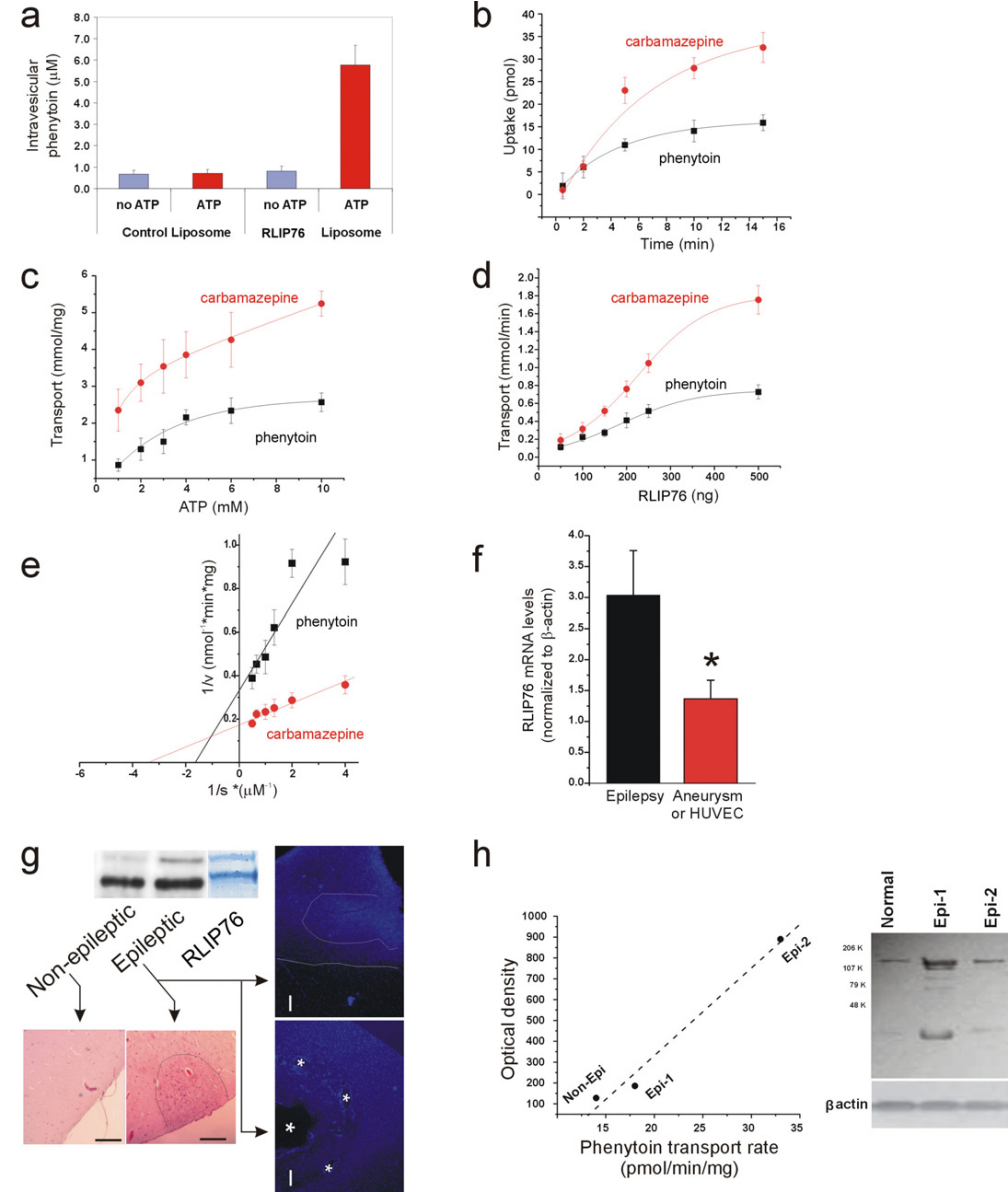
**Phenytoin Transport by RLIP76. A) **Requirement of both ATP and RLIP76 for increased liposomal uptake of ^14^C-PHE. Experiments in a-e were performed four times and triplicate determinations were performed for each data point. **B) **Time dependent uptake of ^14^C-PHE and ^14^C-CBZ by RLIP76-proteoliposomes in the presence of ATP. Data were fitted by y = y_0_+A_1_e^(-x/t)^. **C) **Saturable kinetics of ^14^C-PHE and ^14^C-CBZ transport by purified recombinant human RLIP76 with respect to ATP concentrations. Data were fitted by a single exponential. **D) **Transport of PHE and CBZ by RLIP76-proteoliposomes. Radiolabeled drugs were incubated in the absence or presence of 4 mM ATP with RLIP76 liposomes containing variable amounts of RLIP76. Unless both ATP and RLIP76 were present, drug-uptake was close to the detection limit. Data points were fitted by a Sigmoid y = A_2 _+ (A_1_-A_2_)/(1 + exp(x-x_0_)/dx). **E) **Determination of K_m _values for RLIP76-mediated transport of ^14^C-PHE and ^14^C-CBZ. **F) **RLIP76 mRNA expression in normal (aneurysm, HUVEC) and "epileptic" endothelial cells isolated from tissue resections [31,34]. Note that RLIP76 was significantly (p < 0.05) increased in endothelial cells isolated from multiple drug resistant patients (n = 6) compared to control tissue (n = 8). **G) **Analysis of specimens by Western-blot analysis confirmed these findings. We compared protein expression in dysplastic or normal cortex within the same patient [42,43]. Note that the actively epileptic cortex was characterized by gross abnormality and increased expression of RLIP76. The Coomassie stained band shows the migration pattern of purified human RLIP76 to emphasize the increased levels of signal in both bands. The *arrows *point to histological sections from the same regions used to isolate protein. Note that abnormal clustering of cells is evident on H&E stained sections and in neighboring samples stained with DAPI. The *bars *indicate 100 μm; the *dotted *and *dashed *lines show the extent of abnormal nuclear clustering while the *asterisks *refer to abnormal vascular structure present in these grossly malformed cortices. **H) **RLIP76 is up-regulated in epileptic brain and RLIP76 protein expression levels correlate with transport activity in inside-out membrane vesicles prepared from the same tissues. Data were fitted linearly (R value of 0.99).

Since the predominant endothelial localization of RLIP76 suggested a strategic role in determining multiple drug resistance to antiepileptic drugs, we tested the ability of RLIP76 to extrude the classic antiepileptic drug PHE [28] (Fig. 2). This was examined in an isolated system consisting of asolectin-cholesterol artificial liposomes reconstituted in the presence of purified human RLIP76 [23,24]. Uptake of PHE by liposomes with RLIP76 or without (control) was examined in the presence or absence of ATP. Presence of ATP in the transport medium increased the uptake of ^14^C-PHE by RLIP76-liposomes in a dose dependent fashion, whereas ATP had no effect on uptake by control liposomes (Fig. 2a). We also determined that the intra-vesicular concentration of PHE in control liposomes with or without ATP and in RLIP76 liposomes without ATP were near the extra-vesicular drug-concentration (1 μM), while RLIP76-liposomes in the presence of ATP had intra-vesicular PHE concentrations of 5.7 μM, demonstrating that in the presence of ATP, RLIP76 liposomes are able to concentrate PHE against a gradient, the hallmark of active transport.

Similar findings were obtained when investigating ATP-dependent transport of another classic antiepileptic drug carbamazepine (CBZ). While it is commonly believed that PHE is an MDR1 substrate, uncertainty exists on CBZ extrusion by MDR1 [5,29]. Uptake of PHE or CBZ by inside-out vesicles was a time dependent process with kinetics consistent with a single compartment filling model (Fig. 2b), and the initial velocity of transport could be reasonably estimated by measuring uptake at 2 min after addition of ATP. Both antiepileptic drugs (AED) were transported by RLIP76 liposomes (Fig. 2d) Initial velocity kinetics performed with varying either substrate (ATP or CBZ/PHE), while holding the other constant showed that the K_m _for PHE and CBZ was 0.43 and 0.25 μM respectively (Fig. 2e) and for ATP 1.33 mM (PHE) and 3.3 mM (CBZ) (Fig. 2c).

We then compared function and levels of expression of RLIP76 in non-epileptic brain resected during cerebrovascular surgery unrelated to epileptic pathology or drug resistance vs. epileptic brain. Expression of RLIP76 was determined by Western blot of tissue blocks and mRNA analysis in isolated and cultured brain microvascular endothelial cells (Fig. 2f–h). We found that RLIP76 mRNA expression was greater in epileptic brain or endothelial cells isolated from the same tissue and that RLIP76 protein levels correlated with PHE transport activity measured in inside-out vesicles prepared from brain tissue (Fig. 2h).

**Figure 3:**
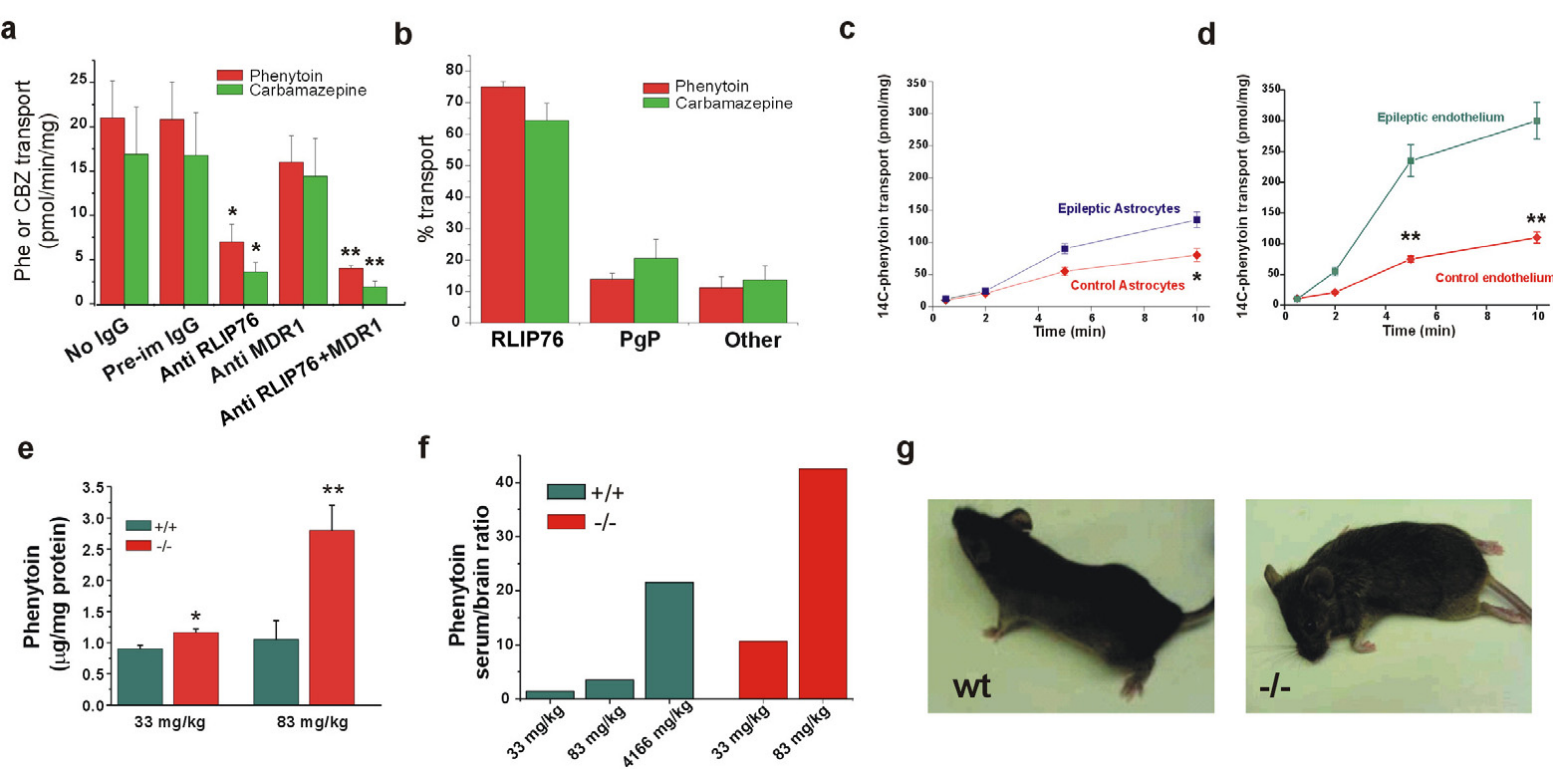
**Phenytoin transport in epileptic brain is mediated by RLIP76. A-B) **Relative contribution of RLIP76 and MDR1 to total AED transport capacity in IOV prepared from brain tissue of non-epileptic, non-multiple drug resistant and multiple drug resistant epileptic patients. This was determined using anti-RLIP76 and anti-MDR1 antibodies [23,25]. In this figure, * and ** represent p < 0.05 and 0.01 respectively. **C-D) **Time dependent PHE uptake by IOV prepared from primary cultures of astrocytes or endothelial cells from normal (diamonds) or epileptic brain (squares). **E-F) **Brain PHE levels in RLIP76^+/+ ^and RLIP76^-/- ^mice 2 h after IP administration of Phosphenytoin at 33 or 83 mg/kg (3 animals/group). **G) **After exposure to the antiepileptic drug, RLIP76^+/+ ^animals (left panel) appeared relatively unaffected compared with RLIP76^-/- ^mice (right panel) where a severe neurological toxicity including extensor posturing, lethargy and status epilepticus were observed.

Functional RLIP76 expression and AED transport activity were further studied in freshly collected human brain samples from epileptic patients. To determine the relative contribution of RLIP76 toward total PHE/CBZ efflux capacity, we examined drug transport in the absence or presence of anti-RLIP76 antibodies [23] in crude membrane vesicles prepared from freshly collected human epileptic brain tissue (n = 8; Fig. 3a, b). Anti-RLIP76 inhibited total transport of PHE and CBZ by 64 ± 6 and 74 ± 1.82% respectively (p < 0.01). When we repeated the same experiments with anti-MDR1 antibodies, we found inhibition by 21 ± 9 (PHE) and 13 ± 1.86 (CBZ) %; exposure to both antibodies resulted in a cumulative inhibition. Interestingly, the amount of PHE extrusion by MDR1 determined by antibody-mediated inactivation was comparable to that described in a previous study after pharmacological blockade by XR9576, a specific MDR1 blocker [30]. These findings indicated that RLIP76 is the predominant AED transporter in brain tissue.

For the above experiments, we directly obtained vesicles from acutely isolated cortical samples. Under these conditions, culture artifacts are avoided but the individual contribution of a given cell type remains undetectable. To determine the cell type involved in the process of RLIP76-mediated PHE extrusion we compared ^14^C-PHE transport in vesicles obtained from endothelial cells and astrocytes isolated and cultured from either control (n = 4) or epileptic (n = 6) brain [31] (Fig. 3c, d). PHE transport was significantly higher in endothelial cells as compared to astrocytes, and greater in both cell types from epileptic tissues. These data are in agreement with the immunocytochemical results showing predominant expression of RLIP76 in human epileptic endothelial cells and not brain parenchymal cells.

PHE has been described as an MDR1 substrate based on experiments performed on knock out mice lacking Pgp [32]. We performed experiments in RLIP76^+/+ ^and RLIP76^-/- ^C57B mice injected i.p. with PHE (33 or 83 mg/kg, 6 animals per group; Fig. 3e). At both doses, brain PHE levels were higher in RLIP76^-/- ^mice as compared with RLIP76^+/+ ^(p < 0.05). However, this was more prominent at the 83 mg/kg level. A statistically significant increase in PHE accumulation in brain was observed at both concentrations (Fig. 3f). RLIP76^-/- ^mice were characterized by higher levels of PHE in both brain and serum compared with the wild-type, consistent with a role of RLIP76 in renal excretion of PHE (not shown). To account for this, a group of wild-type animals was injected with elevated (4166 mg/kg) doses of PHE to achieve serum levels comparable to those seen in knock out animals injected with much lower quantities (83 mg/kg). RLIP76^-/- ^mice demonstrated greater neurotoxicity after administration of PHE; side effects in these animals included lethargy and status-epilepticus (Fig. 3g).

## Conclusion

Taken together, our results show that RLIP76 is an important PHE and CBZ transporter at the human blood brain barrier, and its expression is increased in the BBB from patients with drug-resistant epilepsy. RLIP76 fulfilled many of the predicted properties for a mediator of CNS pharmacoresistance, including: 1) presence at the anatomical interface between brain and blood; 2) transport of the antiepileptic drugs PHE and CBZ; 3) functional expression in brain microvascular endothelial cells but not in parenchymal glia or neurons; and 4) increased CNS accumulation of PHE in RLIP76^-/- ^mice. These results also demonstrate for the first time that the putative mediator of multiple drug resistance in epilepsy, MDR1 [33], is in fact overshadowed in potency by another ATP-dependent transporter. Our results are also in accord with previous reports which questioned the relevance of MDR1 as a multiple drug resistance mechanism, while rather suggesting a role in neuroglial protection [10]. We also confirmed that CBZ is a poor MDR1 substrate [34].

Several important questions remain unanswered. For example, is the widespread distribution of MDR1, MRPs (in particular MRP2 [35]) and RLIP76 in epileptic brain also linked to the pathology itself? Are these transporters expressed as a response to a hostile environment or are they regulated exclusively by chemotherapy? Both MDR1 and MRP are involved in cell survival, and recent evidence by this laboratory [8-10] have shown that apoptotic mechanisms are lacking in epileptic brain. Interestingly, the original proposed role for RLIP76 was indeed that of a molecule involved in detoxification or protection of cells living in hostile environments [36,37]. More recently, Yadav et al. have confirmed a dual role for RLIP76, consisting of anti-apoptotic and drug resistance functions [38]. Thus, purely on the bases of overall function, MDR1 and RLIP76 are indistinguishable. It is possible that multiple drug resistance molecules, in addition to cooperating in drug extrusion, also play a role in the control of cellular homeostasis.

In conclusion, we report a novel, non-ABC transporter mediated mechanism of antiepileptic drug resistance that may synergistically cooperate with MDR1. The relative contribution of each transporter as determined in vitro and ex situ suggests a predominant role for RLIP76. Our results are consistent with a predominant role of the blood-brain barrier in determining multiple drug resistance to antiepileptic drugs.

## Methods

### Reagents

CNBr-activated Sepharose 4B, 1-chloro-2,4-dinitrobenzene (CDNB), PMSF, β-mercaptoethanol (BME), EGTA, EDTA, ATP, butylated hydroxytoluene (BHT), and polidocanol (C_12_E_9_), were purchased from Sigma Chemical Co., St. Louis, MO. DE-52 (diethylaminoethyl cellulose) anion exchanger was purchased from Whatman International Ltd. Maidstone, England. Bio-Beads (SM-2 adsorbent) and Chelex-100 resin were purchased from Bio-Rad Laboratories (Hercules, CA). Tryptone and yeast extract for preparing culture media were purchased from DIFCO laboratories, Detroit, MI. PHE was purchased from Pfizer New York, NY. [4-^14^C]-5,5-diphenylhydantoin (specific activity 49.37 mCi/mmol) and [^14^C]-CBZ (specific activity 22.6 mCi/mmol) were purchased from Perkin Elmer Life Sciences, Boston, MA and Sigma Chemical Co., St. Louis, MO, respectively. Source of anti-RLIP76 IgG used in these studies was the same as previously described [39]. DNP-SG and DNP-SG-Sepharose-4B affinity resin were prepared as previously described [39].

### Tissue procurement and inside-out vesicle preparation (IOV)

Human subjects (see Table 1 for details) were used for these studies as donors of tissue samples. The investigation conforms to the principles outlined in the Declaration of Helsinki. For freshly isolated surgical samples, patient consent was obtained as per the Institutional Review Board instructions before collection of the specimens; autopsy materials were obtained from organ donors. Endothelial cells and glia were isolated from brain specimens from patients undergoing a temporal lobectomy to relieve medically intractable seizures (n = 12) or cortices of patients undergoing surgery for removal of vascular malformations (n = 3). Tissue from autopsy material was used for Fig. 1a. Blood vessels were isolated from resected tissue by manually pulling out a combination of penetrating pial and superficial pial vessels. The methods are described in detail elsewhere [9,34]. The plasma membrane vesicles of human brain cells were prepared as described elsewhere [21,23,25]. Briefly, the cells were separated form the suspension buffer by centrifugation and lysed by incubation in hypotonic buffer (0.5 mM sodium phosphate, pH 7.0, containing 0.1 mM EDTA and 0.1 mM PMSF for 1.5 h, followed by homogenization. After centrifugation of the homogenate at 12,000*g *(10 min at 4°C), the postnuclear supernatant was further centrifuged at 100,000*g *for 40 min at 4°C. The resulting pellet was suspended in the reconstitution buffer (250 mM sucrose-10 mM Tris-HCl, pH 7.4) homogenized with tight fitting Dounce homogenizer and layered over 38% sucrose in 5 mM Hepes-KOH, pH 7.4. After centrifugation at 28,000*g *for 2 h at 4°C the interphases were collected, washed by centrifugation in the reconstitution buffer (100,000*g*), and passed 20 times through a 27-gauge needle for vesicle formation.

### Anti-RLIP76 and anti-MDR1 antibodies

Goat-anti-human Pgp antibody C-19 was purchased from Santa Cruz Biotech, CA. Polyclonal rabbit-anti-RLIP76 IgG as well as pre-immune IgG were prepared and purified as described previously [39,40]. Briefly, recombinant human RLIP76 expressed in *E. coli *and purified by DNP-SG-Sepharose affinity purification as previously described [39] was injected (75 μg) into New Zealand White rabbit after obtaining preimmune serum. After booster doses of 50 μg each at two week intervals, post-immune serum was obtained. The IgG fraction from pre- and post-immunized, heat-inactivated serum was purified by DE-52 anion exchange chromatography, followed by protein-A-Sepharose affinity chromatography. The purity of the antibody was checked by SDS-PAGE as well as Western blotting against goat anti-rabbit IgG. Aliquots of the antibody were stored at -86°C and checked regularly by aerobic and anaerobic cultures for contamination. The specificity of anti-RLIP76 and other antibodies has been stringently established: purified recombinant RLIP76 used for raising polyclonal antibodies was demonstrated to be homogenous by amino acid composition analysis demonstrating amino acid yields within 96% of those expected according to its sequence SELDI-MS demonstrating a pattern of [M+H] peaks consistent with homogenous preparations of RLIP76 [39].

### Immunocytochemistry

To investigate the expression of RLIP76 protein and its localization in both various human tissues and in human epileptic brain, slide mounted sections of 10 μm thickness from frozen brain tissue were labeled as previously described [8,9]. Purified anti-RLIP76 IgG was used as primary antibody. FITC conjugated purified donkey anti-rabbit IgG (Jackson Immunoresearch Laboratories, West Grove, Pennsylvania) was the secondary antibody.

### Preparation of liposomes containing purified recombinant RLIP76

The 1968 bp full length open reading frame cDNA of human RLIP76 was cloned from a λgt11 human bone marrow library by immuno-screening, using anti-DNP-SG ATPase antibodies and was subcloned into the prokaryotic expression vector, pET30a(+) (Novagen, Madison, Wisconsin), creating the pET30-RLIP76 plasmid free of extraneous sequences. This plasmid was transformed into *E. coli *BL21 (DE3). Protein was expressed in *E. coli *BL21 (DE3) grown at 30°C after induction with 0.4 mM IPTG [39]. DNP-SG affinity chromatography was used as described previously [39,40] to obtain purified RLIP76. ATPase activity was performed as previously described to monitor purification [26]. Purity was checked by SDS-PAGE, Western blot analysis and amino acid composition analysis as previously described [39]. Purified protein was dialyzed against liposome reconstitution buffer (10 mM Tris-HCl, pH 7.4, 2 mM MgCl_2_, 1 mM EGTA, 100 mM KCl, 40 mM sucrose, 2.8 mM BME, 0.05 mM BHT, and 0.025% polidocanol). An aqueous emulsion of soybean asolectin (40 mg/ml) and cholesterol (10 mg/ml) was prepared in the reconstitution buffer by sonication. This emulsion was diluted 10 fold by addition of dialyzed RLIP76 in reconstitution buffer to achieve a final RLIP76 concentration of 0.1 mg/ml. The reaction mixture was sonicated at 15 s at 50 W. Vesiculation was initiated by addition of SM-2 Bio-beads (200 mg/ml) pre-equilibrated in the reconstitution buffer (without polidocanol). Vesiculation was carried out for 4 h at 4°C, followed by removal of SM-2 Bio-beads by centrifugation. The vesicles were collected and analyzed for protein content, transport activity, and microbial contamination. Control vesicles to measure non-specific transport, were prepared using an equal amount of crude protein from *E. coli *not expressing RLIP76.

### Transport studies

For transport studies, crude membrane inside-out vesicles (IOV) were prepared from different brain tissues and cells by the method as previously described [23,25]. Briefly, stock solutions of 40 mM MgCl_2 _and 40 mM of ATP were prepared in buffer containing 40 mM sucrose and 10 mM Tris-HCl, pH 7.4. The reaction mixture (120 μL) consisted of IOV protein (80 μg), 10 mM Tris-HCl, pH 7.4, 40 mM sucrose, 4 mM MgCl_2 _and appropriate volume of radio-labeled ^14^C-PHE and ^14^C-CBZ were added to attain a final concentration of 1 μM. To start the reaction, buffer with or without ATP was added to achieve a final concentration of 0 or 4 mM ATP. The uptake was stopped by rapid filtration of a fixed aliquot (30 μL) of the reaction mixture through 96 well nitrocellulose plates (0.45 μm pore size). After filtration, the bottoms of the nitrocellulose membranes were blotted dry with filter paper and punched out, and the associated radioactivity was measured by placing in liquid scintillation counting vials. Scintillation vials were vortexed thoroughly, allowed standing for 1 h at room temperature, and counted in a liquid scintillation counter. Each determination was performed in triplicate. ATP-dependent uptake of ^14^C-PHE and ^14^C-CBZ were determined by subtracting the radioactivity of the control without ATP from that of the experimental containing ATP and the transport was calculated in terms of pmol/min/mg protein.

### cDNA array and mRNA

The details of the tissue culture procedures and mRNA extraction have been described previously [5]. Briefly, surgically obtained specimens were washed in phosphate buffered saline (PBS) and incubated in collagenase type II (2 mg/ml, Worthington chemicals) at 37°C for 20 min to dissociate the endothelial cells. Collagenase was then washed off with the medium used for growing ECs (1.5 g/100 ml, MCDB 105 supplemented with Endothelial Cell Growth Supplement, 15 mg/100 ml, heparin 800 units/100 ml, 10% fetal bovine serum, and penicillin/streptomycin 1%, Sigma chemicals), and ECs were harvested using a sterile cotton swab soaked in the medium. EC stained positive for Von Willebrand factor and were negative for glial fibrillary acidic protein. ECs were purged from the culture dishes by gentle enzymatic dissociation (collagenase) and collected by centrifugation. Equal amounts of the pellet were used for total RNA isolation and protein extraction. Total RNA was extracted with the Trizol reagent (Gibco Labs). Integrity of the isolated RNA was confirmed by agarose formaldehyde gels. For gene expression analysis, human GENEFILTERS™ (Research Genetics Inc., Huntsville, Alabama) were used for this study. Each filter membrane contains approximately 4,000 known human genes. ^33^P-dCTP was used to label probe used for hybridization to produce clean and sharp signals, as recommended by the manufacturer.

### RLIP76 knockout animals

RLIP76^+/- ^heterozygous knockout animals were commissioned from Lexicon genetics, and prepared by the strategy described previously [41]. Briefly, C57B mice (12 wk old), born of *RLIP76*^+/- ^× *RLIP76*^+/- ^mating, were genotyped by PCR strategy. We generated C57B mice which carry heterozygous (+/-) or homozygous (-/-) disruption of the RLIP76 gene, and established colonies of RLIP76+/+, RLIP76+/-, and RLIP76-/- C57B mice by segregation and mating of animals based on genotyping by polymerase chain reaction (PCR) on tail DNA. Western-blot analysis of mouse tissues using anti-RLIP76 antibodies confirmed decreased RLIP76 levels in the RLIP76+/- mouse, and its absence in tissues from the RLIP76-/- mouse [41]. Consistent with the observed function of RLIP76 as a transporter of GS-E and doxorubicin (DOX) in cell culture studies [23,25], GS-E and DOX transport in membrane vesicles was decreased in a stepwise fashion from the RLIP76+/+, to RLIP76+/-, to RLIP76-/- mice [41].

### Measurement of PHE concentration in wild type and RLIP76 knockout mouse serum and brain tissues

Twelve week old C57B mice born of heterozygous × heterozygous mating were genotyped by PCR on mouse tail DNA using forward, reverse and LTR primers [41]. PHE measurements were performed on 5 wild type (RLIP76^+/+^) and 5 RLIP76 knockout (RLIP76^-/-^) animals sacrificed 2 h after a single i.p.-injection of phosphenytoin or PHE. A 10% homogenate of mouse brain tissues was prepared and centrifuged at 28,000 × g for 45 min at 4°C. PHE levels in homogenate and plasma was measured using the Dade Behring Clinical Multichannel Analyzer with the PHE Flex^® ^reagent cartridge, a method based on PETINIA technology.

## Abbreviations used

RALBP1, official human genome designation for human Ral-binding protein-1, synonymous with RALBP1, and homologous to rat RALBP1 and mouse RIP1. We refer to the mouse, rat and human proteins as RLIP76 in the present communication; AED, anti-epileptic drug; BBB, blood-brain barrier; CBZ, carbamazepine; EC, endothelial cells; GSH, glutathione; GS-E, glutathione electrophile conjugates; DOX, doxorubicin; MCDB, modified Czapek Dox broth; PHE, phenytoin.

## Authors' contributions

Designed the study, participated in its design and coordination and helped to draft the manuscript: Sanjay Awasthi, Damir Janigro

Performed pharmacological experiments, including knock out animal studies: Sharad S. Singhal

Contributed to designing of pharmacological experiments and assisted in the writing of the discussion section: Yogesh C. Awasthi

Performed immunocytochemical, mRNA, and Western blotting analysis: Kerri L Hallene, Vince Fazio, Luca Cucullo, Gabriele Dini

## Figures and Tables

**Table 1:** Human Tissue Donor Information

**ID**	**Age**	**Sex**	**Notes**	**AED failed**	**Use**
1	0.8	M	EPi- Hemispherectomy	None	WB
2	0.3	M	Epi-Hemispherectomy	Ketogenic Diet	WB, AED transport studies
3	57	F	Temporal Lobe Epilepsy	PHE, CBZ,TPM	WB
4	33	F	Control (AVM)	None	WB
5	61	F	Temporal Lobe epilepsy	VAL,CBZ,TPB	WB, AED transport studies
6	30	M	Control Parasaggital Cist	None	WB, AED transport studies
7	0.9	F	Frontal/Parietal Epilepsy	VAL, PBT, TPM	WB
8	14	M	Temporal Lobe Epilepsy	CBZ, Keppra, PBT,TPM, VAL	WB
9	42	M	Temporal Lobe Epilepsy	VAL, PBT, primidone, tiagabine,	AED transport studies
10	27	F	Temporal Lobe Epilepsy	PHE,VAL,gabapentin,CBZ, TPM	AED transport studies
11	7	M	R. Lateral Parietal/Occipital/Temporal Lobes	PBT, PHE, TPM, CBZ, DZP, LMT	AED transport studies
12	14	M	Temporal Lobe Epilepsy	VAL,Keppra, CBZ, other	AED transport studies
13	31	F	Temporal Lobe Epilepsy	gabapentin, CBZ,VAL,PHE,PBT	AED transport studies
14	60	F	Temporal Lobe Epilepsy	PBT, MBL, TGL, DPT, VLM, MSL, FBT	In vitro cells AED
15	43	F	Focal Epi	N/A	In vitro cells AED
16	56	M	Focal Epi	PHE, PBT, CBZ, VAL, ETS	In vitro cells AED
17	43	F	Temporal Lobe Epilepsy	PHE, CBZ, VAL, GBP	mRNA analysis
18	40	F	Control (Aneurysm)	NONE	mRNA analysis
20	58	F	Focal Epi	PHE, PBT, VAL, PRM, FBT, GBP, TPM	mRNA analysis
21	35	M	Temporal Lobe Epilepsy	PHE, CBZ, VAL, PRM, VGB	mRNA analysis
22	28	M	Temporal Lobe Epilepsy	PHE, PBT, CBZ, VAL, TPM, CZP, GBP	mRNA analysis
23	38	M	Temporal Lobe Epilepsy	PHE, CBZ, VAL, FBM, GBP	mRNA analysis
24	39	M	Temporal Lobe Epilepsy	PHE, PBT, VAL, PRM, MSD	mRNA analysis
25	31	M	Temporal Lobe Epilepsy	PHE, PBT, VAL, TPM	mRNA analysis
26	23	M	Temporal Lobe Epilepsy	PHE, PBT, CBZ, VAL, GBP	mRNA analysis
27	45	F	Control (Aneurysm)	NONE	mRNA analysis
28	22	F	Control (Aneurysm)	NONE	mRNA analysis
36	7	M	Temporal Lobe Epilepsy	PHE, CBZ, VAL, GBP	ICC
37	28	F	Temporal Lobe Epilepsy	PHE, PBT, CBZ, VAL	ICC
38	7	M	Temporal Lobe Epilepsy	?	ICC
39	4	F	Frontal and Parietal Epilepsy	PHE, CBZ, VAL, PRM,	ICC
40	8	F	Temporal lobe epilepsy	CBZ, PHE, VAL	ICC
41	24	M	Control (AVM)	NONE	ICC
42	1	M	Frontal Epilepsy	CBZ, PHE, VAL	ICC
43	25	M	Temporal Lobe Epilepsy	Phe CBZ	ICC
44	1	M	Hemispherectomy	None	ICC
45	23	F	Contol (AVM)	None	ICC
46	23	M	Temporal lobe epilepsy	gabapentin, CBZ,VAL,PHE,	ICC
47	23	M	Control (AVM)	None	ICC
48	7	M	TSC	?	ICC
49	3	F	Epilepsy Right Hemi	?	ICC
50			Autopsy		ICC
51			Autopsy		ICC
52			Autopsy		ICC
53			Autopsy		ICC
54			Autopsy		ICC,WB
55			Autopsy		ICC,WB

## References

[B1] Sander JW (2002). The problem of the drug-resistant epilepsies. Novartis Found Symp.

[B2] Sisodiya SM (2003). Mechanisms of antiepileptic drug resistance. Curr Opin Neurol.

[B3] Begley DJ (2004). ABC transporters and the blood-brain barrier. Curr Pharm Des.

[B4] Schinkel AH, Wagenaar E, Mol CA, van Deemter L (1996). P-glycoprotein in the blood-brain barrier of mice influences the brain penetration and pharmacological activity of many drugs. J Clin Invest.

[B5] Owen A, Pirmohamed M, Tettey JN, Morgan P, Chadwick D, Park BK (2001). Carbamazepine is not a substrate for P-glycoprotein. Br J Clin Pharmacol.

[B6] Weiss J, Kerpen CJ, Lindenmaier H, Dormann SM, Haefeli WE (2003). Interaction of antiepileptic drugs with human P-glycoprotein in vitro. J Pharmacol Exp Ther.

[B7] Doran A, Obach RS, Smith BJ, Hosea NA, Becker S, Callegari E, Chen C, Chen X, Choo E, Cianfrogna J, Cox LM, Gibbs JP, Gibbs MA, Hatch H, Hop CE, Kasman IN, Laperle J, Liu J, Liu X, Logman M, Maclin D, Nedza FM, Nelson F, Olson E, Rahematpura S, Raunig D, Rogers S, Schmidt K, Spracklin DK, Szewc M, Troutman M, Tseng E, Tu M, Van Deusen JW, Venkatakrishnan K, Walens G, Wang EQ, Wong D, Yasgar AS, Zhang C (2005). The impact of P-glycoprotein on the disposition of drugs targeted for indications of the central nervous system: evaluation using the MDR1A/1B knockout mouse model. Drug Metab Dispos.

[B8] Marroni M, Marchi N, Cucullo L, Abbott NJ, Signorelli K, Janigro D (2003). Vascular and parenchymal mechanisms in multiple drug resistance: a lesson from human epilepsy. Curr Drug Targets.

[B9] Marroni M, Agarwal M, Kight K, Hallene K, Hossain M, Cucullo L, Signorelli K, Namura S, Janigro D (2003). Relationship between expression of multiple drug resistance proteins and p53 tumor suppressor gene proteins in human brain astrocytes. Neuroscience.

[B10] Marchi N, Hallene KL, Kight KM, Cucullo L, Moeddel G, Dini G, Bingaman W, Vezzani A, Janigro D (2004). Significance of MDR1 and multiple drug resistance in refractory human epileptic brain. BMC Med.

[B11] Sills GJ, Mohanraj R, Butler E, McCrindle S, Collier L, Wilson EA, Brodie MJ (2005). Lack of association between the C3435T polymorphism in the human multidrug resistance (MDR1) gene and response to antiepileptic drug treatment. Epilepsia.

[B12] Kwan P, Brodie MJ (2005). Potential role of drug transporters in the pathogenesis of medically intractable epilepsy. Epilepsia.

[B13] Maines LW, Antonetti DA, Wolpert EB, Smith CD (2005). Evaluation of the role of P-glycoprotein in the uptake of paroxetine, clozapine, phenytoin and carbamazapine by bovine retinal endothelial cells. Neuropharmacology.

[B14] Park SH, Weinberg RA (1995). A putative effector of Ral has homology to Rho/Rac GTPase activating proteins. Oncogene.

[B15] Jullien-Flores V, Mahe Y, Mirey G, Leprince C, Meunier-Bisceuil B, Sorkin A, Camonis JH (2000). RLIP76, an effector of the GTPase Ral, interacts with the AP2 complex: involvement of the Ral pathway in receptor endocytosis. J Cell Sci.

[B16] Cantor SB, Urano T, Feig LA (1995). Identification and characterization of Ral-binding protein 1, a potential downstream target of Ral GTPases. Mol Cell Biol.

[B17] Jullien-Flores V, Dorseuil O, Romero F, Letourneur F, Saragosti S, Berger R, Tavitian A, Gacon G, Camonis JH (1995). Bridging Ral GTPase to Rho pathways. RLIP76, a Ral effector with CDC42/Rac GTPase-activating protein activity. J Biol Chem.

[B18] Quaroni A, Paul EC (1999). Cytocentrin is a Ral-binding protein involved in the assembly and function of the mitotic apparatus. J Cell Sci.

[B19] Rosse C, L'Hoste S, Offner N, Picard A, Camonis J (2003). RLIP, an effector of the Ral GTPases, is a platform for Cdk1 to phosphorylate epsin during the switch off of endocytosis in mitosis. J Biol Chem.

[B20] Sharma R, Sharma A, Yang Y, Awasthi S, Singhal SS, Zimniak P, Awasthi YC (2002). Functional reconstitution of Ral-binding GTPase activating protein, RLIP76, in proteoliposomes catalyzing ATP-dependent transport of glutathione conjugate of 4-hydroxynonenal. Acta Biochim Pol.

[B21] Sharma R, Singhal SS, Cheng J, Yang Y, Sharma A, Zimniak P, Awasthi S, Awasthi YC (2001). RLIP76 is the major ATP-dependent transporter of glutathione-conjugates and doxorubicin in human erythrocytes. Arch Biochem Biophys.

[B22] Sharma R, Singhal SS, Wickramarachchi D, Awasthi YC, Awasthi S (2004). RLIP76 (RALBP1)-mediated transport of leukotriene C4 (LTC4) in cancer cells: implications in drug resistance. Int J Cancer.

[B23] Awasthi S, Singhal SS, Singhal J, Yang Y, Zimniak P, Awasthi YC (2003). Role of RLIP76 in lung cancer doxorubicin resistance: III. Anti-RLIP76 antibodies trigger apoptosis in lung cancer cells and synergistically increase doxorubicin cytotoxicity. Int J Oncol.

[B24] Awasthi S, Singhal SS, Sharma R, Zimniak P, Awasthi YC (2003). Transport of glutathione conjugates and chemotherapeutic drugs by RLIP76 (RALBP1): a novel link between G-protein and tyrosine kinase signaling and drug resistance. Int J Cancer.

[B25] Awasthi S, Singhal SS, Singhal J, Cheng J, Zimniak P, Awasthi YC (2003). Role of RLIP76 in lung cancer doxorubicin resistance: II. Doxorubicin transport in lung cancer by RLIP76. Int J Oncol.

[B26] Singhal SS, Singhal J, Sharma R, Singh SV, Zimniak P, Awasthi YC, Awasthi S (2003). Role of RLIP76 in lung cancer doxorubicin resistance: I. The ATPase activity of RLIP76 correlates with doxorubicin and 4-hydroxynonenal resistance in lung cancer cells. Int J Oncol.

[B27] Stuckler D, Singhal J, Singhal SS, Yadav S, Awasthi YC, Awasthi S (2005). RLIP76 transports vinorelbine and mediates drug resistance in non-small cell lung cancer. Cancer Res.

[B28] Treiman DM, Woodbury DM, Levy RH, Mattson RH and Meldrum BS (1998). Absorption, distribution and excretion of phenytoin.

[B29] Potschka H, Fedrowitz M, Loscher W (2001). P-glycoprotein and multidrug resistance-associated protein are involved in the regulation of extracellular levels of the major antiepileptic drug carbamazepine in the brain. Neuroreport.

[B30] Martin C, Berridge G, Mistry P, Higgins C, Charlton P, Callaghan R (1999). The molecular interaction of the high affinity reversal agent XR9576 with P-glycoprotein. Br J Pharmacol.

[B31] Marroni M, Kight KM, Hossain M, Cucullo L, Desai SY, Janigro D (2003). Dynamic in vitro model of the blood-brain barrier. Gene profiling using cDNA microarray analysis. Methods Mol Med.

[B32] Schinkel AH (1999). P-Glycoprotein, a gatekeeper in the blood-brain barrier. Adv Drug Deliv Rev.

[B33] Abbott NJ, Khan EU, Rollinson CM, Reichel A, Janigro D, Dombrowski SM, Dobbie MS, Begley DJ (2002). Drug resistance in epilepsy: the role of the blood-brain barrier. Novartis Found Symp.

[B34] Dombrowski S, Desai S, Marroni M, Cucullo L, Bingaman W, Mayberg MR, Bengez L, Janigro D (2001). Overexpression of multiple drug resistance genes in endothelial cells from patients with refractory epilepsy. Epilepsia.

[B35] Potschka H, Fedrowitz M, Loscher W (2003). Multidrug resistance protein MRP2 contributes to blood-brain barrier function and restricts antiepileptic drug activity. J Pharmacol Exp Ther.

[B36] Awasthi S, Sharma R, Yang Y, Singhal SS, Pikula S, Bandorowicz-Pikula J, Singh SV, Zimniak P, Awasthi YC (2002). Transport functions and physiological significance of 76 kDa Ral-binding GTPase activating protein (RLIP76). Acta Biochim Pol.

[B37] Awasthi YC, Sharma R, Cheng JZ, Yang Y, Sharma A, Singhal SS, Awasthi S (2003). Role of 4-hydroxynonenal in stress-mediated apoptosis signaling. Mol Aspects Med.

[B38] Yadav S, Zajac E, Singhal SS, Singhal J, Drake K, Awasthi YC, Awasthi S (2005). POB1 over-expression inhibits RLIP76-mediated transport of glutathione-conjugates, drugs and promotes apoptosis. Biochem Biophys Res Commun.

[B39] Awasthi S, Cheng J, Singhal SS, Saini MK, Pandya U, Pikula S, Bandorowicz-Pikula J, Singh SV, Zimniak P, Awasthi YC (2000). Novel function of human RLIP76: ATP-dependent transport of glutathione conjugates and doxorubicin. Biochemistry.

[B40] Singhal SS, Singhal J, Cheng J, Pikula S, Sharma R, Zimniak P, Awasthi YC, Awasthi S (2001). Purification and functional reconstitution of intact ral-binding Gtpase activating protein, RLIP76, in artificial liposomes. Acta Biochim Pol.

[B41] Awasthi S, Singhal SS, Yadav S, Singhal J, Drake K, Nadkar A, Zajac E, Wickramarachchi D, Rowe N, Yacoub A, Boor P, Dwivedi S, Dent P, Jarman WE, John B, Awasthi YC (2005). RLIP76 is a major determinant of radiation sensitivity. Cancer Res.

[B42] Boonyapisit K, Najm I, Klem G, Ying Z, Burrier C, LaPresto E, Nair D, Bingaman W, Prayson R, Luders H (2003). Epileptogenicity of focal malformations due to abnormal cortical development: direct electrocorticographic-histopathologic correlations. Epilepsia.

[B43] Marusic P, Najm IM, Ying Z, Prayson R, Rona S, Nair D, Hadar E, Kotagal P, Bej MD, Wyllie E, Bingaman W, Luders H (2002). Focal cortical dysplasias in eloquent cortex: functional characteristics and correlation with MRI and histopathologic changes. Epilepsia.

